# Stress type–specific small extracellular vesicle signatures reflect divergent biological responses to acute psychosocial and physical challenges

**DOI:** 10.1038/s41598-025-21575-5

**Published:** 2025-10-09

**Authors:** Dirk A. Moser, Tobias Tertel, Fabian Berg, Elisabeth M. Hummel, Petra Platen, Bernd Giebel, Robert Kumsta

**Affiliations:** 1 Department of Genetic Psychology, Faculty of Psychology, Ruhr-University Bochum, Universitätsstraße 150, 44801 Bochum, Germany; 2 Institute for Transfusion Medicine, University Hospital Essen, University of Duisburg-Essen, Essen, Germany; 3 Department of Sports Medicine & Sports Nutrition, Faculty of Sport Science, Ruhr-University Bochum, Gesundheitscampus-Nord 10, 44801 Bochum, Germany; 4 Department of Behavioural and Cognitive Sciences, Laboratory for Stress and Gene-Environment Interplay, University of Luxemburg, Porte Des Sciences, L-4366 Esch-Sur-Alzette, Luxembourg

**Keywords:** Biomarkers, Neuroscience, Psychology, Psychology

## Abstract

Maladaptive stress responses are associated with a variety of psychological and physical disorders, often characterized by molecular indicators of dysregulated stress pathways. Small extracellular vesicles (sEVs), which play a key role in intercellular communication, may be critically involved in these processes. In this study, we quantified sEV concentrations (specifically CD9^+^, CD63^+^, and CD81^+^ markers) in the plasma of twenty young, healthy men before and after exposure to both acute psychosocial and physical stress, using imaging flow cytometry (IFCM). Both stressors induced significant increases in cortisol, catecholamines, and cell-free DNA (cfDNA) in all participants. sEVs, however, were elevated only after physical stress, showing a rapid rise particularly in CD9- and CD63-positive vesicles, followed by a return to baseline within 40 min. In contrast, psychosocial stress elicited highly heterogeneous, non-directional sEV responses. Recursive partitioning further revealed clearly distinct sEV patterns for psychosocial and physical stress, with the highest discriminatory power observed for CD44⁺ sEVs. These findings support the existence of stressor-specific sEV signatures that could serve as future biomarkers of individual stress experience and act as effectors of distinct signal transduction pathways in both psychosocial and physical stress.

**Supplementary Information:**

The online version contains supplementary material available at 10.1038/s41598-025-21575-5.

## Introduction

Stress is a global burden with significant psychological, physiological, and economic consequences. In Western industrialized nations alone, stress-related costs exceed 200 billion USD annually, reflecting its association with numerous physical and mental disorders^1^. Despite its profound impact, the molecular pathways linking stress to disease remain incompletely understood, underscoring the need for deeper insights into stress-induced physiological processes to inform effective interventions.

Since the foundational work of Cannon and Selye^2–4^, the field of molecular stress physiology has evolved substantially. A particularly promising area of emerging research focuses on extracellular vesicles (EVs)—including small extracellular vesicles (sEVs) in the exosome size range (70–150 nm^5,6^), microvesicles (100–1000 nm^7,8^) and apoptotic vesicles (< 1 µm to > 5 µm^9–11^). These vesicles are critical mediators of intercellular communication, enabling targeted signaling across short and long distances. Exosomes originate as intraluminal vesicles (ILVs) within multivesicular endosomes (MVEs) and are released upon fusion with the plasma membrane^12^. In contrast, microvesicles and apoptotic vesicles are formed by direct budding from the plasma membrane or fragmentation of dying cells, respectively.

It is now evident that virtually all cells release EVs via distinct mechanisms, and EVs have emerged as central players in indirect intercellular communication. However, distinguishing between EV subtypes of similar size remains a major challenge. Although many EV preparations exhibit biological activity, some vesicles may primarily function in cellular waste disposal, transporting non-processable byproducts to clearance organs such as the liver or kidneys. While we are not yet able to reliably distinguish EVs based on function, their cell type–specific molecular signatures make them promising biomarkers and tools for probing complex physiological responses.

Due to methodological limitations, current isolation techniques cannot reliably distinguish exosomes from other sEVs, necessitating the analysis of heterogeneous populations. These vesicles carry lipids, proteins, and nucleic acids such as mRNA and miRNA—initially considered exclusive to exosomes^13–15^. More recent findings show that sEV subtypes differ in composition: some carry exosomal proteins but lack RNA, while others transport RNA with few exosomal surface markers^16^. These observations underscore the complexity of sEV biology and suggest that surface proteins, particularly integrins, may regulate interactions with target cells^17,18^.

Because stress triggers complex systemic responses that involve both heightened metabolic demands and precise intercellular communication, sEVs plausibly play a central role orchestrating these adaptive processes. The brain, though constituting only about 2% of total body weight, accounts for roughly 20% of the body’s total energy consumption^19,20^. This immense energy demand not only reflects the brain’s continuous processing and integration of internal and external stimuli but may also support its role in coordinating systemic responses to stress via sEV-mediated signaling. Similarly, skeletal muscle, comprising approximately 40% of body mass, undergoes substantial metabolic reprogramming during physical exertion and stress^21^. The high energy turnover in both tissues suggests that part of their metabolic budget may be allocated to the production and release of sEVs, which serve as metabolically costly but highly efficient vehicles for systemic communication^22,23^. This mechanism could allow the brain and muscles to modulate peripheral physiological systems in a stressor-specific and temporally precise manner, thus enhancing the organism’s capacity to adapt to diverse psychosocial and physical challenges^24–31^.

Crucially, sEVs can traverse the blood–brain barrier bidirectionally^32^, facilitating a dynamic exchange of molecular information between the central nervous system and peripheral tissues. Given the distinct emotional and physiological signatures of psychosocial versus physical stress, sEVs may carry stressor-specific molecular cargo that fine-tunes metabolic and behavioral responses. This positions them not merely as by-products of cellular activity, but as active mediators in psychophysiological regulation and stress adaptation.

Although EV dynamics have been studied in the context of physical exertion and stress-related diseases^24,33–43^, direct comparisons of sEV responses to acute psychosocial versus physical stress in healthy individuals are lacking. To address this gap, we re-analyzed plasma samples from a previous study^44^, collected before and after psychosocial (Trier Social Stress Test^45^) and physical stress (treadmill ergometry^46^). In that work, plasma cortisol, adrenaline, noradrenaline, and cfDNA increased under both stress conditions before gradually returning toward baseline. However, the Social Emotional Response Scale (SERS) revealed distinct emotional patterns: “Tense Arousal” dominated after physical stress, while “Self-Directed Emotions” and “Anxiety” were predominant following psychosocial stress. Since conventional biomarkers did not capture these differences in individual stress perception, we explored sEVs as potential novel indicators of stress processing using imaging flow cytometry (IFCM^47–52^), which allows sensitive, high-throughput detection and characterization of sEV subpopulations at the single-vesicle level.

We conducted a pilot study with model character, analyzing plasma from 20 healthy young men at five time points before and after stress exposure (–2, + 2, + 15, + 30, + 40 min) using a validated panel of 23 antibodies (Table 1) primarily targeting hematopoietic sEVs. We hypothesized that stress-induced changes—particularly in platelet-derived sEVs—could serve as early biomarkers, given their known increase after physical activity. Psychosocial stress-induced cortisol elevations might similarly modulate blood cell sEV release. Both stress types, despite differing in intensity and physiological demands (e.g., oxygen consumption, heart rate, lactate), activate the hypothalamic–pituitary–adrenal axis (HPA-axis) and sympathetic nervous system, leading to hormone-driven modulation of sEV release and composition. Our focus was to identify both, distinct sEV subpopulations—“ExerVs” for physical exertion and “PsychEVs” for psychosocial stress—and a shared population, “StressEVs,” responsive to both modalities.

**Table 1:** Information on the antibodies.

Antibody	Conjugate	Clone	Order Nr	Company	Isotyp	Volume
CTLA-4	PE	BNI3	555,853	BD Bioscience	mouse IgG2a, κ	0.10
CD9	PE	MEM-61	1P-208-T100	EXBIO	mouse IgG1, κ	0.25
CD13	PE	QA19A12	111,003	BioLegend	rat IgG2a, κ	0.25
CD14	APC	REA599	130–110-520	Miltenyi	human IgG1, rec	0.10
CD16	PE	3G8	555,407	BD Bioscience	mouse IgG1, κ	0.25
CD24	PE	M1/69	130–102-732	Miltenyi	rat IgG2bκ	0.25
CD41	AF488	MEM-06	A4-309-T100	EXBIO	mouse IgG1, κ	0.25
CD44	APC	MEM-85	1A-221-T100	EXBIO	mouse IgG2a, κ	0.25
CD61	FITC	SZ21	IM1758	Beckman Coulter	mouse IgG1, κ	0.25
CD63	APC	MEM-259	1A-343-T100	EXBIO	mouse IgG1, κ	0.25
CD66b	APC	6/40c	-*	LeukoCom	mouse IgG1, κ	0.10
CD81	FITC	JS64	B25329	Beckman Coulter	mouse IgG2a, κ	0.25
CD82	PE	ASL-24	342,104	BioLegend	mouse IgG1, κ	0.25
CD90	APC	5,00E + 10	1A-652-T100	EXBIO	mouse IgG1, κ	0.10
CD100	PE	133-1C6	1P-772-T100	EXBIO	mouse IgM, κ	0.25
CD171	AF647	L1-OV198.5	371,607	BioLegend	mouse IgG2a, κ	0.10
CD206	BV421	15.2	321,126	BioLegend	mouse IgG1, κ	0.10
CD227	FITC	HMPV	559,774	BD Bioscience	mouse IgG1, κ	0.25
CD235a	APC	GA-R2	551,336	BD Bioscience	mouse IgG2a, κ	0.25
HLA-ABC	FITC	B9.12.1	IM1838U	Beckman Coulter	mouse IgG2a, κ	0.25
HLA-DR	ECD	Immu-357	B92438	Beckman Coulter	mouse IgG1, κ	0.25
PD-L1	PE	29E.2A3	329,705	BioLegend	mouse IgG2a, κ	0.25
PS	AF488	1H6	16–256	Sigma-Aldrich	mouse IgG1, κ	0.10

Listed above are all antibodies used in this study, including conjugate, clone, catalog number, manufacturer, isotype, and dilution.

Specifically, we (i) assessed the day-to-day stability of plasma sEV expression under baseline conditions; (ii) analyzed acute stress-induced changes in sEV profiles using IFCM; and (iii) applied a classification model, including machine learning, to test whether our antibody panel could reliably distinguish stress types. By characterizing these responses, we aimed to advance understanding of systemic stress adaptation and identify candidate biomarkers for stress-related disorders.

## Material and methods

### Experimental model and subject details

Participants (n = 20) were healthy male sport science students, aged between 18 and 36 years (mean = 23.3 ± 3.8 (SD)), with a normal body mass index (mean = 23.4 ± 1.5). They had no history of or current mental health disorders, no chronic or acute physical illnesses, and were not taking any medications or drugs at the time of the study. Participants refrained from exercise for 24 h before testing and consumed a standardized breakfast on the morning of the tests. As a pilot study, only male participants were recruited to minimize confounding variables and eliminate potential effects related to the female menstrual cycle. Furthermore, all stress tests were conducted at either 9 a.m. or 11 a.m. to minimize cortisol diurnal cycle variations and potential influence of lunchtime food intake. All participants provided written informed consent prior to participation. The study was approved by the local ethics committee of the Faculty of Psychology at Ruhr University Bochum (reference number 153/2014) and conducted in accordance with the Declaration of Helsinki.

### Method details

Participants were exposed to acute psychosocial and physical stressors in a randomized order on separate days. Stress inductions and testing were spaced at least 2 days apart. Half of the participants completed the TSST first, while the other half began with the exercise protocol. Testing order was assigned pseudo-randomly. Upon arrival, participants completed a Physical Activity Readiness Questionnaire (PAR-Q^53^), reviewed by a sports physician, and had a venous catheter inserted 45 min before stress induction. After completing questionnaires for 25 min, participants remained in a dedicated, separate room until the onset of the stress protocol. This procedure ensured a calm environment and helped reduce anticipatory stress prior to collection of the first blood sample. Blood and saliva samples were collected before, and at 2-, 15-, 30-, and 40-min post-stress. Participants completed the Social Emotional Response Scale (SERS) at four time points (-2, + 2, + 15, and + 30 min), rating their arousal, self-directed emotions, and anxiety on a scale from 1 (not at all) to 4 (a lot).

### Induction of psychosocial stress

Psychosocial stress was induced using the Trier Social Stress Test (TSST), which includes preparation, free speech, and an unanticipated math task in front of judges and a camera. The TSST reliably activates the hypothalamic–pituitary–adrenal (HPA) axis, causing significant cortisol elevation due to the uncontrollability and social evaluative threat elements^45,54^.

### Induction of physical exercise stress

To induce physical stress, participants underwent an exhaustive treadmill exercise protocol designed to roughly match the duration of the TSST (15 min). The protocol began with a 5-min warm-up walk at 1.0 m/s on a treadmill with a 15% incline. Thereafter, the speed increased by 0.2 m/s every 30 s until the participant reached subjective exhaustion, at which point the treadmill was stopped. Under the selected conditions, participants completed the treadmill exercise within a range of 11 min 46 s ± 1 min 41 s.

### Plasma preparation and cfDNA quantification

Five milliliters of whole blood were collected at each time point in EDTA collection tubes (EDTA Monovettes, Sarstedt, Germany) and immediately centrifuged at 1600 × g for 10 min at 4 °C. The plasma was transferred to a fresh tube and subjected to a second centrifugation for 10 min at 16,000 × g at 4 °C. Subsequently, the plasma was filtered through a 0.8 μm filter, and aliquots were stored at − 80 °C until further analysis.

cfDNA was extracted from 0.9 mL of plasma using the QIAamp Circulating Nucleic Acid Kit (Qiagen, Hilden, Germany), widely regarded as the gold standard for cfDNA extraction, following the manufacturer’s protocol. The elution was performed in a final volume of 100 μl of H_2_O.

### Quantitative PCR (qPCR) and Hormonal Analysis

Cell-free DNA (cfDNA) was quantified by qPCR, as previously described^44^. Plasma cortisol was measured using a commercial ELISA (Demeditec, Kiel, Germany), and catecholamines (adrenaline and noradrenaline) were determined by high-performance liquid chromatography (HPLC) at the Laboratory for Stress Monitoring (LSM, Göttingen, Germany) following a solvent extraction protocol adapted from Smedes et al.^55^. Full methodological details are provided in reference^44^.

### Imaging flow cytometry (IFCM)

To analyze PsychEVs, ExerVs, and StressEVs, an imaging flow cytometry (IFCM) approach was employed using the Amnis ImageStreamX Mk II (Luminex, USA). IFCM combines flow cytometry’s throughput with microscopy’s precision, allowing high-resolution single-vesicle analysis of sEVs. Plasma samples from the cfDNA study frozen at − 80 °C were again centrifuged after thawing at 10.000 × g for 10 min at 4 °C to remove cryoprecipitates. The supernatant was transferred to a fresh tube, and 10 µL aliquots of plasma were incubated with 10 µL of fluorochrome-conjugated antibodies targeting EV markers diluted in PBS, as indicated in Table 1. Samples were incubated at room temperature for 1 h in the dark to ensure specific labeling, followed by a dilution in PBS to 100 µL final volume. Controls—antibody-only, buffer-only, isotype, and detergent-treated—ensured background assessment per MIFlowCyt-EV standards^56^. The ImageStreamX Mk II operated at 60 × magnification with a low flow rate (0.3795 ± 0.0003 μL/min), acquiring data over 5 min per well to optimize single-particle detection. Fluorescence and scatter plots were used to gate EV populations, quantify concentrations, and validate labeling efficiency^50,51^. Data were processed using IDEAS 6.2 software (Luminex), employing customized masks and spot-counting features to identify and quantify individual sEVs. Fluorescence and scatter plots were used to gate populations based on labeling intensity and side scatter, and EV concentrations were calculated. This high-resolution IFCM approach allowed precise EV subpopulation analysis, revealing profiles linked to psychosocial and physical stress. In addition, performing IFCM adheres to the Minimal Information for Studies of Extracellular Vesicles (MISEV) guidelines^56–58^, which recommend strict quantification, imaging, and molecular characterization. Contrary to widespread assumptions, transmission electron microscopy (TEM), Western blotting (WB), and nanoparticle tracking analysis (NTA) are not mandatory, as they often lack the resolution needed for precise characterization.

### Statistical analysis

All statistical analyses were performed using R Studio (version 2024.12.1 + 563). To minimize the risk of false-positive findings, the number of statistical tests was reduced to those necessary to address the study’s primary aims.

To ensure comparability of baseline sEV levels between psychosocial and physical stress conditions, Bonferroni-corrected paired t-tests were conducted for all 23 sEV subpopulations. Moreover, we assessed the total variance that can be attributed to differences between individuals by calculating the intraclass correlation (ICC) of a null model and a model that included stress types as a fixed effect. Subsequently, repeated-measures analyses of variance (rmANOVA) were applied to test for time × stress-type interactions for each sEV marker. If a significant interaction was observed, follow-up paired t-tests (baseline vs. + 2, + 15, + 30, and + 45 min) were performed. Bonferroni correction was used to adjust for multiple comparisons.

To assess the potential of sEV profiles discriminating between stress types, a classification model based on recursive partitioning was employed (rpart package, complexity parameter = 0.05, tenfold cross-validation)^59^. Half of the data from both stress conditions was randomly selected for model build and training (*n* = 20), while the remaining half served as the testing set (*n* = 20). Stress conditions were alternated across folds to avoid bias. The full sEV panel across all time points was used as input for the model.

Outlier removal was conducted prior to all parametric tests. For each combination of stress type and time point (2 × 5 = 10 per marker), the median and Median Absolute Deviation (MAD) were calculated. Values outside the range of median ± 3 × MAD were considered outliers and removed^60^. To preserve the integrity of within-subject comparisons, participants with missing values were excluded from the respective test. However, all available data were retained for classification analysis.

Normality assumptions were assessed using Shapiro–Wilk tests for each marker across the 10 condition × time point combinations. Due to violations, CD61, CD81, and CD100 underwent square root transformation to better meet test assumptions. CD9, CD44, and CD171 did not require transformation. However, all other markers were ln-transformed. Transformation was applied selectively to preserve interpretability where possible.

All data generated during this study are provided in Supplemental Data File S3. The R script used for the analysis is available in Supplemental Data File S4.

## Results

### Psychosocial as well as physical stress leads to significant increases in cortisol, catecholamines, and cell-free DNA

This study aimed to investigate the effects of acute psychosocial and physical stress on sEV release. We explored the differential response of sEV populations, measured by 23 different sEV markers, to better understand the distinct physiological mechanisms activated by each stress type. Each participant provided blood samples at multiple time points: before stress exposure (baseline), and at 2-, 15-, 30-, and 40-min post-stress. As previously reported in detail^61^, both stress tests led to significant increases in plasma concentrations of cortisol, adrenaline/noradrenaline, and circulating cell-free nucleic acids in all participants (Fig. S1).

### Baseline levels of sEV markers are highly reproducible

**Fig. 1:**
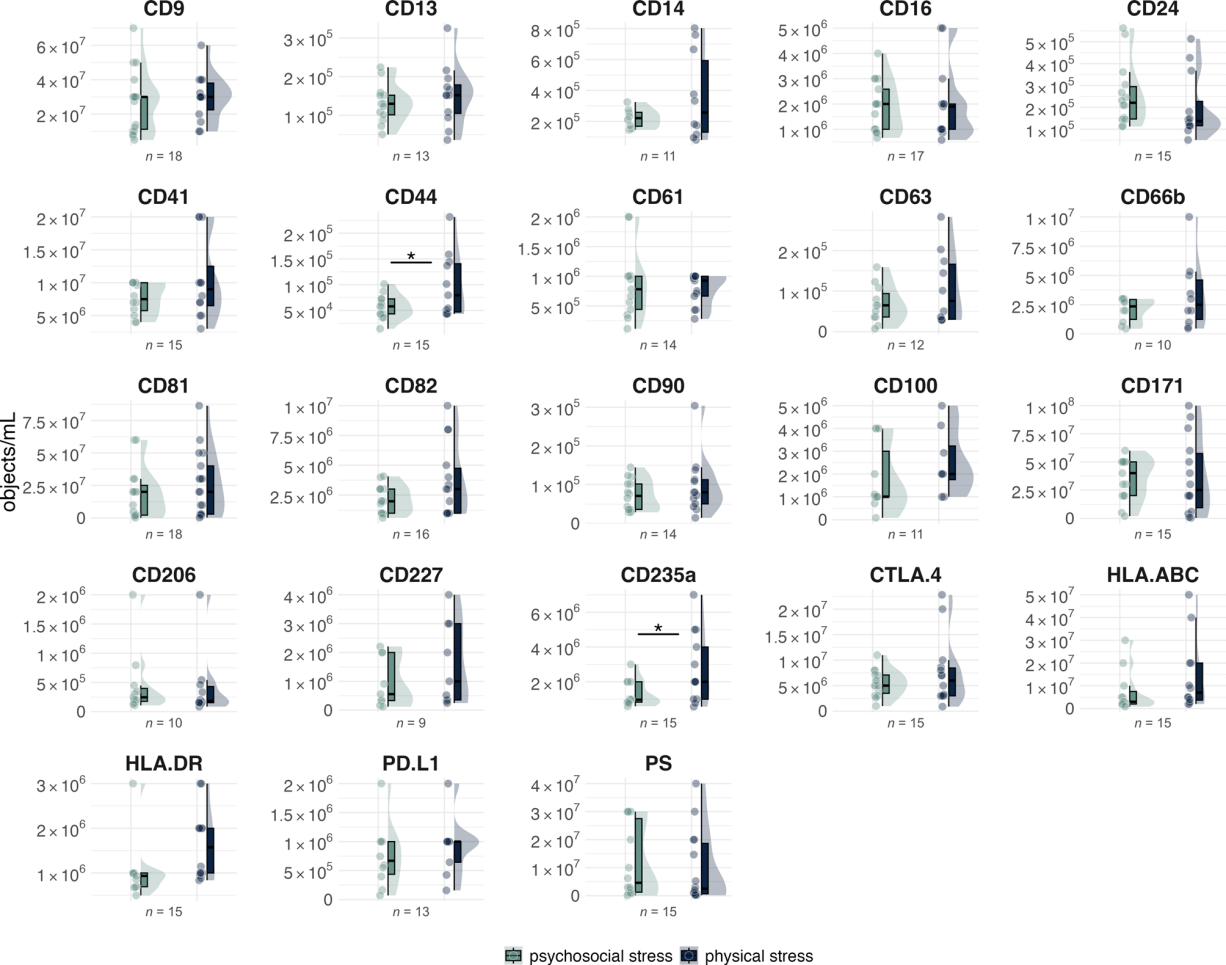
Comparison of sEV levels at baseline. Individual sEV plasma concentrations, expressed as [objects/mL], and distribution curves are shown for the sEV panel at baseline (–2 min). Sample sizes vary across sEVs due to the exclusion of outliers and unpaired observations, enabling paired t-test analysis. Across the study, the 23 sEV populations exhibit largely stable and comparable baseline abundances between psychosocial and physical stress conditions. Even for CD44⁺ and CD235a⁺ sEVs, which show statistically significant differences, the distribution curves substantially overlap. Notably, differences in plasma concentrations across individual sEV types are reflected in distinct y-axis scales. p < 0.05.

To evaluate the stability and suitability of the selected sEV markers as stress-related biomarkers, we first assessed inter- and intra-individual variation in baseline plasma levels across both study sessions. Establishing baseline stability is essential to ensure that observed changes reflect stress-induced dynamics rather than day-to-day variability. Among the 23 surface markers analyzed, 21 showed no significant differences between the two baseline conditions (psychosocial vs. physical stress), indicating high reproducibility of marker detection across experimental days (Fig. 1). CD44⁺ (t(14) = -2.42, p = 0.03, d = -0.62, 95% CI [-1.41, -0.16]) and CD235a⁺ sEVs (t(14) = -2.36, p = 0.03, d = -0.61, 95% CI [-1.20, -0.13]) were significantly different, although their distribution curves showed substantial overlap, suggesting that these minor deviations may reflect normal variation rather than biologically meaningful shifts. Overall, plasma sEV profiles at baseline were largely consistent across both conditions. Consistently, calculation of ICC (ρ = 0.01, 95% CI [0, 0.04]) revealed negligible variance between testing sessions and suggests random error rather than systematic differences between individuals as a source of total variance.

### CD9^+^, CD41^+^, and CD81^+^ sEVs dominate the plasma sEV profile

Next, we quantified the absolute plasma concentrations of each sEV subset based on marker expression, reported in objects per milliliter of plasma. CD13⁺, CD14⁺, CD24⁺, CD44⁺, CD63⁺, and CD90⁺ sEVs were typically found at concentrations around 10^5^ objects/mL. Intermediate levels (~ 10⁶ objects/mL) were observed for CD16⁺, CD61⁺, CD66b⁺, CD82⁺, CD100⁺, CD206⁺, CD227⁺, CD235a⁺, HLA-DR⁺, and PD-L1⁺ sEVs. The most abundant marker-positive sEV subsets included CD9⁺, CD41⁺, CD81⁺, CD171⁺, CTLA-4⁺, HLA-ABC⁺, and PS⁺ sEVs, often exceeding 10⁷ objects/mL. These findings highlight the pronounced heterogeneity in sEV abundance, with tetraspanin-positive (CD9⁺, CD81⁺) and platelet-derived (CD41⁺) vesicles consistently constituting the most abundant subsets.

### CD13^+^, CD14^+^, and CD41^+^ sEVs increase transiently after physical stress

**Fig. 2:**
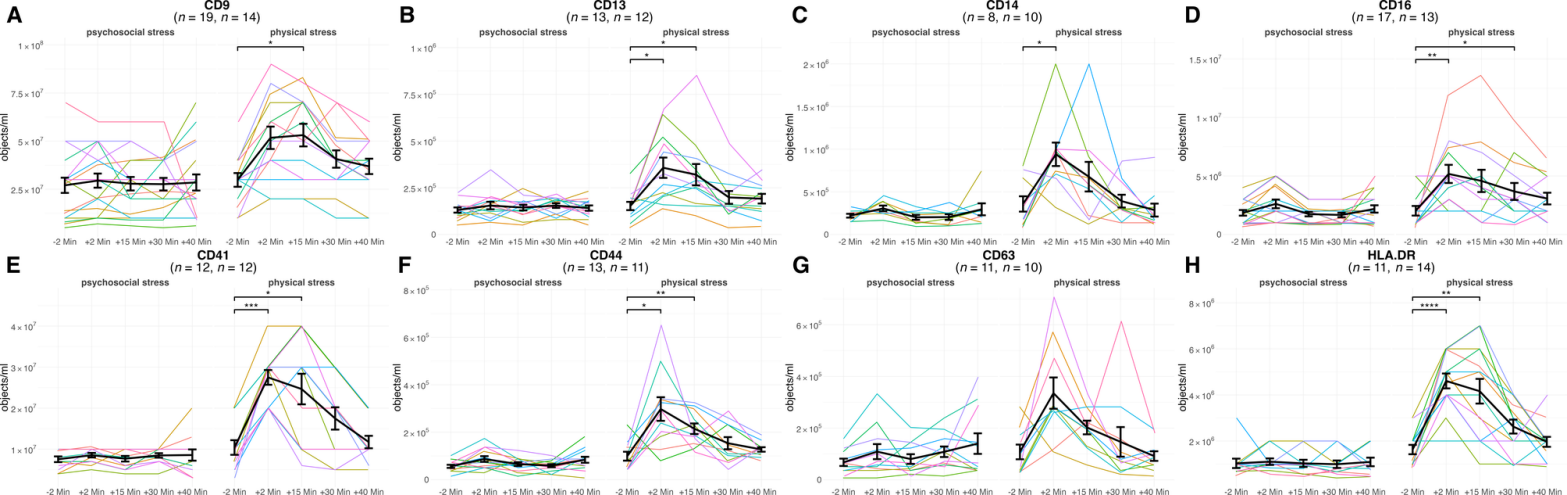
Pairwise comparison of sEVs with a significant time × stress type interaction. Panels A-H show plasma sEV levels measured as [objects/mL] over the course of the experiment. Colored lines represent the intra-individual trajectories of plasma sEV levels, while black lines indicate mean values. Error bars represent the standard error of the mean (SEM). Data are shown for all subjects with available plasma measurements at all time points for at least one stress condition, after removal of outliers. Only sEVs showing a significant time × stress type interaction are presented and tested for significant changes from baseline, to reduce the number of statistical comparisons. Note that the overall abundance of different sEVs in plasma is heterogeneous, as reflected in the varying y-axes across the panels. Significance: p < 0.05 (), p < 0.01 (), p < 0.001 (), p < 0.0001 (**).

We next investigated whether acute stress exposure induced dynamic changes in sEV subpopulations over time and whether such responses differed between psychosocial and physical stress. Repeated-measures ANOVA identified significant interaction effects between time and stress type for eight markers: CD9, CD13, CD14, CD16, CD41, CD44, CD63, and HLA-DR. Follow-up Bonferroni-corrected paired t-tests indicated that CD13⁺ (aminopeptidase N, associated with myeloid lineage), CD14⁺ (monocytic), CD16⁺ (commonly associated with NK cells and non-classical monocytes), CD41⁺ (platelet-derived), CD44⁺ (expressed on mesenchymal stem cells, activated T cells, and monocytes), and HLA-DR⁺ (antigen-presenting/activated cells) sEVs significantly increased at + 2 min after physical stress (p < 0.05; Fig. 2). Several markers remained elevated beyond this point: CD9⁺, CD13⁺, CD41⁺, CD44⁺, and HLA-DR⁺ sEVs at + 15 min, and CD16⁺ at + 30 min. CD63⁺ sEVs showed a numerical increase at + 2 min (p = 0.058), though this did not reach statistical significance. Likewise, none of these markers did reach statistical significance in response to psychosocial stress. Full statistical details are presented in Table S1. The remaining 15 markers, which did not yield significant interaction effects, are shown descriptively in Fig. S2.

These findings suggest that physical stress elicits a reproducible, short-term release of specific sEV subpopulations—predominantly derived from platelets and myeloid-lineage immune cells—whereas psychosocial stress results in more heterogeneous and less consistent sEV dynamics.

### CD44^+^ sEV concentrations enable classification of stress types

**Fig. 3:**
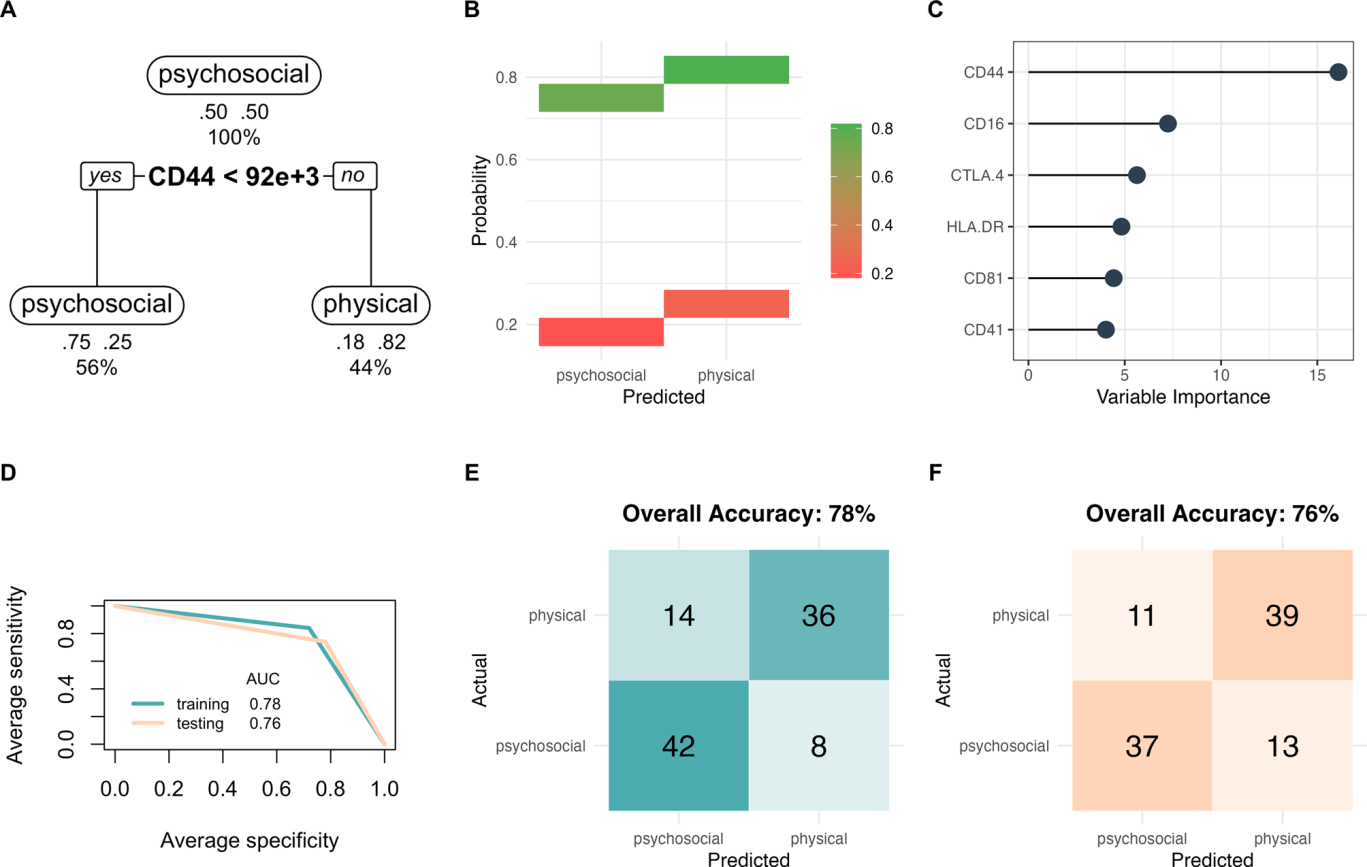
Recursive partitioning classification of psychosocial and physical stress. (**A**) Decision tree of the classification analysis using plasma levels of all 23 sEV markers across 5 time points and both stress types from the training dataset as input. The training dataset consisted of precisely half the data available. (**B**) Probabilities of the class prediction show that psychosocial and physical stress were correctly assigned in 75% and 82% of the cases (green) and wrongly assigned in 25% and 18% of the cases (red). (**C**) Importance of all variables influencing the classification analysis and building a sum of 100% suggest likewise to the decision tree that CD44 was most important in distinguishing stress types. (**D**) ROC curve presenting the sensitivity, specificity and AUC of the classification for both halves of the data. (**E**, **F**) confusion matrix of the training and testing dataset presenting the accuracy of predicting stress types based on the sEV recursive partitioning classification model.

Lastly, we assessed the suitability of the sEV panel as a whole discriminating between the two stress types. Therefore, we applied a classification analysis using recursive partitioning that explored whether plasma sEV profiles could distinguish between psychosocial and physical stress responses. The model was build using 50% (*n* = 20) of all 23 marker-positive sEV subpopulations across five time points and both conditions. Within the training set, the model classified 56% of observations as psychosocial and 44% as physical stress (Fig. 3A). The initial split was based on CD44⁺ sEV concentration, with a threshold of 9.2 × 10^4^ objects/mL identified as most discriminative.

Probabilities for correct assignment reached 75% for psychosocial and 82% for physical stress (Fig. 3B). CD44⁺ sEVs showed the highest variable importance, followed by CD16⁺, CTLA-4⁺, HLA-DR⁺, CD81⁺, and CD41⁺ subsets (Fig. 3C). ROC curve analysis revealed an area under the curve (AUC) of 0.78 in the training and 0.76 in the testing dataset (Fig. 3D). Sensitivity and specificity were 0.84 and 0.72 in the training set, and 0.74 and 0.78 in the test set, respectively. Overall classification accuracy exceeded 76% across both datasets (Fig. 3E,F), indicating preliminary but promising classification outcome. Hence, these findings show that the physiological response to psychosocial and physical stress is related to distinguishable profiles of hematopoietic sEV markers.

## Discussion

The relationship between stress and health has long been a focus of scientific inquiry, particularly regarding the distinct physiological responses triggered by different stressors. Small extracellular vesicles have recently gained recognition as both mediators and potential biomarkers of stress-related processes, reflecting the underlying molecular mechanisms of adaptation and systemic communication. In this study, we analyzed plasma sEV profiles in healthy young men following acute psychosocial and physical stress, using a within-subject design and high-resolution single-vesicle analysis via imaging flow cytometry (IFCM).

We first examined the baseline stability of plasma sEVs across different days. Most sEV markers showed stable baseline concentrations across test days, underscoring their potential as reliable stress-related biomarkers. This temporal consistency aligns with preclinical data on circadian regulation of EV release in animal models^62^. Although CD44- and CD235a-positive vesicles showed baseline differences potentially reflecting anticipatory stress (see supplementary SERS-related material in^44^), as indicated by elevated anxiety scores before the psychosocial stressor, their increases occurred only prior to the physical stressor, suggesting other factors regulate individual sEVs throughout the day. While corresponding human data remain limited, our findings indicate that at least a subset of sEV markers maintain sufficient stability to support longitudinal monitoring in clinical contexts.

The stress-related analyses revealed that physical stress triggered rapid and reproducible increases in several sEV subtypes, including CD13⁺, CD14⁺, CD41⁺, CD44⁺, and HLA-DR⁺ vesicles—predominantly associated with cells of myeloid (e.g., monocytes) and platelet origin. The analyzed hormones and cell-free DNA exhibited significant elevations following both stressors, followed by a rapid homeostatic return to baseline^44^ (Fig. S1). These changes were most pronounced within the first 15 to 30 min post-stress. In contrast, sEVs displayed this consistent response pattern only after physical stress. Following psychosocial stress, however, sEV profiles displayed pronounced alterations, marked by substantial interindividual variability and an absence of a consistent return to baseline at the group level. Such variability likely reflects complex, individualized stress-processing mechanisms rather than a lack of biological responsiveness and is consistent with previous findings linking extracellular vesicles to mental health and psychological stress^36,37,39,63–65^.

We further observed significant post-exercise increases in sEVs carrying epitopes for CD9, CD13, CD14, CD16, CD41, CD44, CD63, and HLA-DR, indicating contributions from multiple cellular sources. CD14 and CD16 point toward monocyte and myeloid-lineage origins, with CD16 also present on certain NK-cells and granulocytes. CD41 reflects platelet derivation, consistent with exercise-induced platelet activation, while CD44—expressed on lymphocytes, monocytes, and endothelial cells—suggests involvement of diverse immune and vascular compartments. CD13, a membrane metalloprotease abundant on myeloid cells, endothelial cells, and some epithelial cells, may indicate vesicle release from cells engaged in immune–vascular interaction. The tetraspanins CD9 and CD63, while broadly expressed, confirm the vesicular nature of the particles and may represent release from multiple cell types. HLA-DR, as a component of MHC-II, points toward antigen-presenting cells such as monocytes, dendritic cells, and activated B cells.

Taken together, these markers extend our prior identification of lymphocyte (CD4, CD8), monocyte (CD14), platelet (CD41, CD42, CD62P), endothelial (CD105, CD146), and antigen-presenting cell (MHC-II) origins for exercise-induced vesicles (ExerVs), underscoring a heterogeneous vesicle pool within the circulation that likely participates in exercise-related signalling and inter-tissue communication. Acute physical exercise can trigger platelet activation through increased vascular shear stress and sympathetic nervous system stimulation, even in the absence of vascular injury, leading to the shedding of vesicles enriched in platelet markers such as CD41, CD61, and CD62P. This process is further amplified by catecholamines (epinephrine, norepinephrine) acting on platelet adrenergic receptors, and by cortisol, which may modulate platelet reactivity over longer periods. Platelet-derived sEVs can carry pro-inflammatory mediators, interact with immune cells, and contribute to immune modulation, partly via platelet–immune cell aggregates. Consistent with other human studies^34^ these platelet-derived vesicles peak immediately after strenuous exercise and typically normalize within an hour, often transporting adhesion molecules, coagulation factors, and bioactive lipids. This profile supports their role in hemostatic preparedness, vascular adaptation, and immune communication, reflecting an evolutionarily conserved “fight-or-flight” mechanism.

A classification model based on CD44⁺ and other responsive sEV subtypes achieved moderate separation between stressor types (AUC 0.76–0.78). CD44⁺ vesicles, expressed by various immune and stromal cells and known to regulate cell adhesion and migration during inflammation, emerged as the most informative feature, alongside CD16⁺, CTLA-4⁺, and HLA-DR⁺ vesicles. While these results are promising, they should be interpreted with caution due to the limited training set, the risk of overfitting and the need for supporting data on the diurnal stability of each marker. Notably, the model’s performance appeared to be primarily driven by the more consistent sEV responses observed after physical stress.

Our antibody panel focused on sEVs of hematopoietic origin, which represent a major component of the circulating EV pool and are responsive to immune and vascular activation. The predominance of CD9⁺, CD81⁺, and CD41⁺ vesicles align with earlier reports from healthy plasma donors^66^, suggesting that tetraspanin- and platelet-derived sEVs dominate the basal plasma profile. In contrast, CD63⁺ vesicles were less abundant, possibly reflecting differences in biogenesis or clearance. The observed increases in CD13⁺, CD14⁺, and HLA-DR⁺ vesicles are consistent with vesicle release from myeloid immune cells such as monocytes or dendritic cells, although definitive assignment to cell types requires co-staining or cargo-based analyses.

Methodologically, IFCM enabled direct quantification of surface marker-positive sEVs in minimally processed plasma, preserving physiological composition and allowing robust time-resolved analyses. This is a key advantage for in vivo studies of dynamic EV release. However, the lack of co-expression data limits resolution of EV subtypes and restricts inferences about cellular origin. Future studies should incorporate multiplexed antibody panels (e.g., multi-channel IFCM or barcoding approaches) and platform-independent workflows to support diagnostic applicability.

However, several limitations must be acknowledged. The male-only cohort limits generalizability, and the 40-min observation window may have missed delayed sEV responses. The absence of molecular cargo profiling restricts insight into functional content. While the sample size aligns with prior EV stress studies^24,27,34,40,41,43,67^, it remains modest and increases the risk of overfitting. These findings should therefore be interpreted with caution. External validation in larger, more diverse cohorts will be essential to confirm the observed patterns and assess their translational relevance. Future studies should also refine classification models using independent datasets and optimized feature selection strategies.

In summary, our data demonstrate that acute physical stress reliably triggers the release of distinct sEV subtypes, particularly platelet- and immune-derived vesicles. In contrast, psychosocial stress elicits more heterogeneous responses. Plasma sEV profiling may thus offer novel insights into stress physiology and, if validated, could support individualized diagnostics in stress-related disorders. In particular, the concepts of psychosocial stress-associated vesicles (PsychEVs) and generalized stress-responsive vesicles (StressEVs) warrant further investigation and validation in future studies.

## Supplementary Information

Supplementary Information 1.

Supplementary Information 2.

Supplementary Information 3.

Supplementary Information 4.

Supplementary Information 5.

## Data Availability

All data generated during this study are provided in Supplemental Data File S3. The R script used for the analysis is available in Supplemental Data File S4.
