# Supplementary material for: Stress type–specific small extracellular vesicle signatures reflect divergent biological responses to acute psychosocial and physical challenges
Source: Sci Rep. 2025 Oct 9;15:35231. doi: 10.1038/s41598-025-21575-5 (PMC12511347; doi:10.1038/s41598-025-21575-5)
Supplement: Supplementary file 1 — Supplementary Information 1. [file 41598_2025_21575_MOESM1_ESM.pdf]

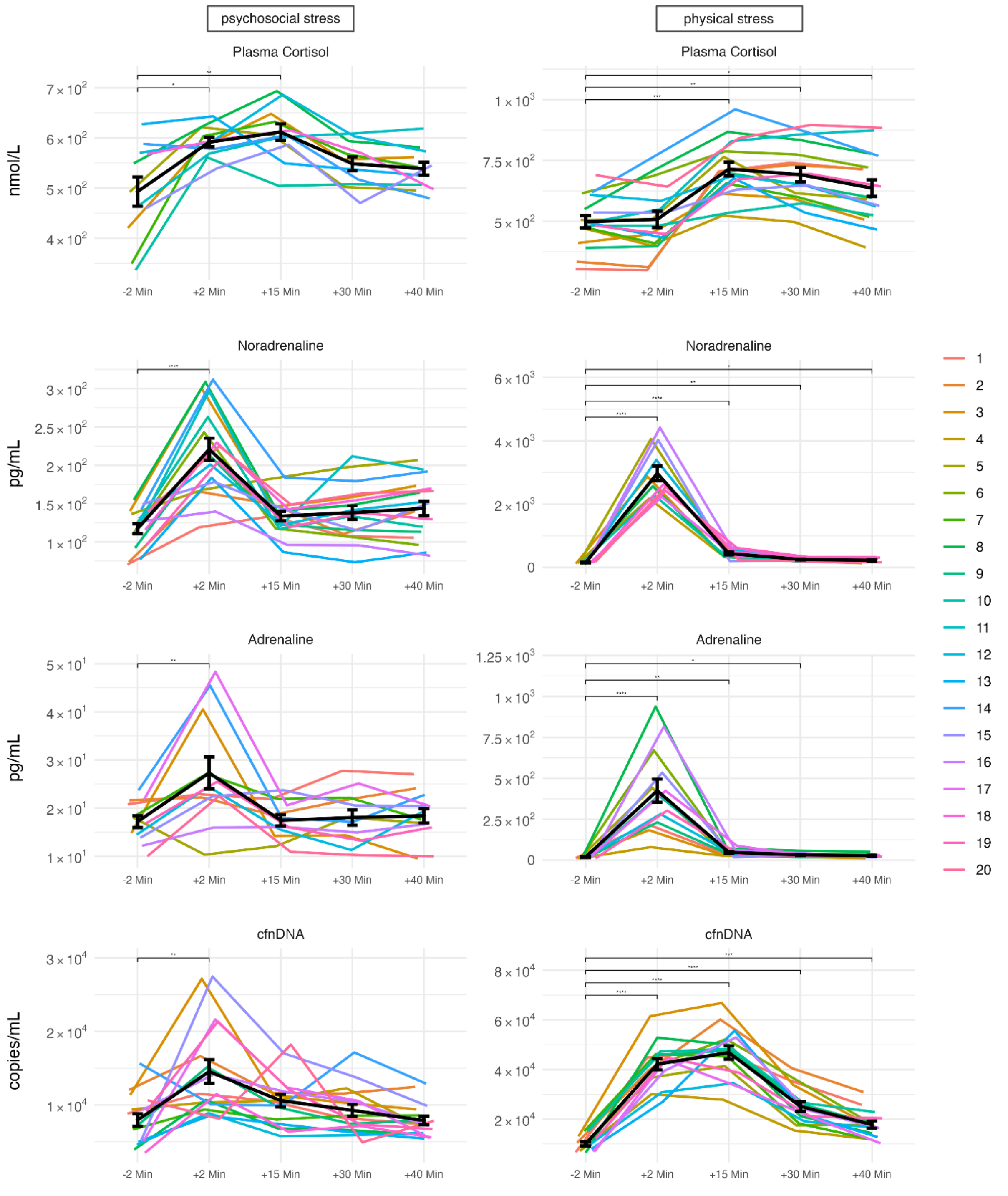

Supplementary Figure S1: Pairwise comparison of Cortisol, Adrenalin/Noradrenaline, and cfnDNA in plasma before and after psychosocial and physical stress

The values of all 20 participants are represented by differently colored curves for all time points before and after psychosocial and physical stress. The black curve represents the means of all participants at each experimental time point, including standard deviations. sEVs given in objects/mL; Adrenaline/Noradrenaline; Plasma cortisol in nmol/L; cfDNA in copies/mL. Detailed information on the statistical analysis can be found elsewhere.
