# Supplementary material for: Stress type–specific small extracellular vesicle signatures reflect divergent biological responses to acute psychosocial and physical challenges
Source: Sci Rep. 2025 Oct 9;15:35231. doi: 10.1038/s41598-025-21575-5 (PMC12511347; doi:10.1038/s41598-025-21575-5)
Supplement: Supplementary file 2 — Supplementary Information 2. [file 41598_2025_21575_MOESM2_ESM.pdf]

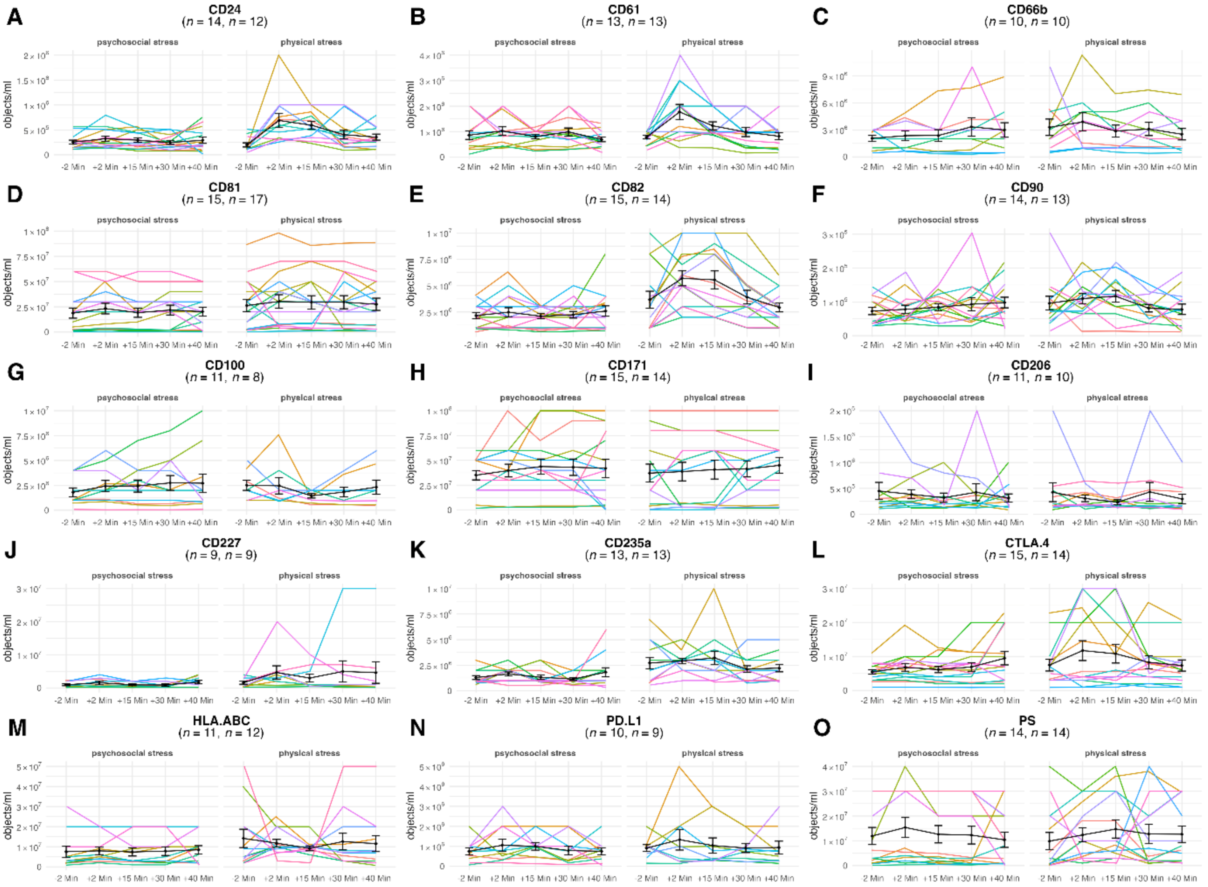

***Supplementary Figure S2**: Plasma levels and time course of the remaining 15 sEVs*

Panels A–O descriptively display the abundance of 15 plasma sEV markers, measured in [objects/mL], over the course of the experiment. No statistical post hoc tests were conducted for these markers, as repeated-measures ANOVA revealed no significant time × stress type interactions. Colored lines indicate intra-individual trajectories of sEV levels, while black lines represent group means. Error bars show the standard error of the mean (SEM). Note that although the y-axis is consistent within each facet plot, it varies across sEVs due to the generally heterogeneous plasma concentrations.
